# Liberty Enlightening the World

**Published:** 1885-06

**Authors:** 


### Liberty Enlightening the World.

This new Wonder of the World, which is now being loaded on the French transport Isere for shipment to this country, is the largest statue in the world. Some idea of its magnitude may be obtained from the fact that forty persons found standing-room within the head. A six-foot man standing on the level of the lips only just reached the eyebrow. While workmen were employed on the crown of her head they seemed to be making a huge sugar-caldron, and they jumped with ease in and out the tip of the nose. Fifteen people might sit round the flame of the torch, which elevation can be reached by a spiral staircase within the outstretched arm.

The London *Daily News*, in speaking of it, says: "It is out and away the largest statue of modern times. The Colossus of Rhodes was nothing to it. It could carry the 'Bravaria' or the 'Hermann' in its arms. It towers to the skies from the yard of the Rue de Chazelles, where it has been eight years in construction, and the view from its coronet sweeps clear of the six-story houses and beyond the walls of Paris."

The weight of this stupendous statue is 440,000 pounds, of which 176,000 pounds are copper and the remainder wrought iron. It is expected to arrive in New York about the 25th of May, where it will be erected on Bedloe's Island, this being the location selected for it by Gen. W. T. Sherman, who was appointed by the President to make the selection. When placed in position it will loom up 305 feet above tide-water, the height of the statue being 151.2 feet, that of the pedestal 91 feet, and foundation 52.10 feet.

This imposing statue, higher than the enormous towers of the great Brooklyn Bridge or the steeple of Trinity Church, which is the loftiest in the city of New York,—higher, in fact, than any of the colossal statues of antiquity,—by its rare artistic proportions, as well as by its stupendous dimensions, will add another to the Wonders of the world. A word should be said of its artistic merit. The pose, stride, and gesture, with its classic face, are pronounced perfect; the drapery is both massive and fine, and in some parts is as delicate and silky in effect as if wrought with a fine chisel on the smallest scale.

The conception and execution of this great work are due to the great French sculptor, M. Bartholdi, who has devoted eight years of his life and most of his fortune to this great work, and whose generous impulses, which must be on a scale commensurate with this noble work, prompted him to make such a gift to the United States. The committee in charge of the construction of the base and pedestal for the reception of this great work are in want of funds for its completion, and have prepared a miniature statuette, an exact counterpart of the original, six inches in height, the figure being made of bronze, the pedestal of nickel silver, which they are now delivering to subscribers throughout the United States for the small sum of $1 each. Aside from its being a lasting souvenir of this colossal statue, it will ornament our homes and bear testimony that we have contributed to the completion of one of the grandest works of modern times. All remittances should be addressed to Richard Butler, Secretary American Committee of the Statue of Liberty, No. 33 Mercer Street, New York. The committee are also prepared to furnish a model, in same metals, twelve inches in height, at $5 each, delivered.

We feel assured our people will be only too eager to testify their grateful sense of the frendliness of this magnanimous offer on the part of the French people, and to reciprocate the kindly and liberal sentiments in which it originated, by thus aiding in an active prosecution of the labors that may be required to give the statue an appropriate base and pedestal. Now is the time to do it. Whoever wishes to have the honor and pleasure of contributing to the erection of the grandest statue of any age, to say nothing of the sentiment that should be welcomed and encouraged, must act promptly, for the money will be raised as sure as the sun rises. Every subscriber sending $1 will be supplied with a miniature counterpart of this great and imperishable statue of "Liberty Enlightening the World."

